# Cortical morphometric changes associated with completeness, level, and duration of spinal cord injury in humans: A case–control study

**DOI:** 10.1002/brb3.2037

**Published:** 2021-01-13

**Authors:** Yun Guo, Feng Gao, Hua Guo, Weiyong Yu, Zhenbo Chen, Mingliang Yang, Degang Yang, Liangjie Du, Jianjun Li

**Affiliations:** ^1^ School of Rehabilitation Medicine Capital Medical University Beijing China; ^2^ Department of Spinal and Neural Functional Reconstruction China Rehabilitation Research Center Beijing China; ^3^ China Rehabilitation Science Institute Beijing China; ^4^ Center of Neural Injury and Repair Beijing Institute for Brain Disorders Beijing China; ^5^ Beijing Key Laboratory of Neural Injury and Rehabilitation Beijing China; ^6^ Department of Rehabilitation Medicine Beijing Tsinghua Changgung Hospital Beijing China; ^7^ Department of Biomedical Engineering Center for Biomedical Imaging Research School of Medicine Tsinghua University Beijing China; ^8^ Department of Radiology China Rehabilitation Research Center Beijing China

**Keywords:** cortical morphometric, gray matter volume, spinal cord injury, voxel‐based morphometry

## Abstract

**Objective:**

This study investigated how the injury completeness, level, and duration of spinal cord injury (SCI) affect cortical morphometric changes in humans.

**Methods:**

T1‐weighted images were acquired from 59 SCI patients and 37 healthy controls. Voxel‐based morphometry analyses of the gray matter volume (GMV) were performed between SCI patients and healthy controls, complete SCI and incomplete SCI, and tetraplegia and paraplegia. Correlation analyses were performed to explore the associations between GMV and clinical variables in SCI patients.

**Results:**

Compared to healthy controls, SCI patients showed decreased GMV in bilateral middle frontal gyrus, left superior frontal gyrus (SFG), left medial frontal gyrus in the whole‐brain analysis, while increased GMV in right supplementary motor area and right pallidum in ROI analysis. The complete SCI had lower GMV in left primary somatosensory cortex (S1) and higher GMV in left primary motor cortex compared with incomplete SCI. Lower GMV was identified in left thalamus and SFG in tetraplegia than that in paraplegia. Moreover, time since injury was positive with the GMV in right pallidum, positive correlations were observed between the GMV in bilateral S1 and total motor and sensory scores, whereas the GMV in left cuneus was negatively correlated with total motor and sensory scores in SCI patients.

**Conclusions:**

The study provided imaging evidence for identifying cerebral structural abnormalities in SCI patients and significant differences in complete/incomplete and paraplegia/tetraplegia subgroups. These results suggested brain structural changes occur after SCI and these changes may depend on the injury completeness, level, and duration.

## Graphical abstract

Our study provided imaging evidence for identifying cerebral structural abnormalities in patients with SCI and significant differences in complete/incomplete and paraplegia/tetraplegia subgroups.

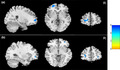

## 1 INTRODUCTION

Spinal cord injury (SCI) is a debilitating and devastating condition that has considerable effects on sensorimotor function. Patients typically suffer from complete or incomplete, tetraplegia or paraplegia based on their completeness of injury and lesion locations. Unfortunately, there is no highly effective disease‐modifying treatment to re‐establish functional neuronal connections (Dietz & Fouad, 2014). Although neurons in the sensorimotor system are not directly damaged due to SCI, the pathways that mediate efferent and afferent information flow between the brain and spinal cord are severely disrupted (Freund et al., 2011). Basic researches have shown significant atrophic changes in the size or number of neurons in the sensorimotor systems after spinal cord transection (Hains et al., 2003; Kim et al., 2006). Brain anatomical atrophy in the white and gray matter or brain abnormal plasticity after SCI might have the potential to reduce the effectiveness of sensorimotor function recovery (Cramer et al., 2005; Freund et al., 2013; Hou et al., 2014; Wrigley et al., 2009). Therefore, it is important to understand the mechanism of brain structural changes and explore their effects on clinical variables for the development of evidence‐based rehabilitation therapy (Kramer et al., 2012).

To date, few structural studies have investigated SCI‐related neuroanatomical changes in humans, but with various and inconsistent results (Chen et al., 2017; Crawley et al., 2004; Freund et al., 2011; Hou et al., 2014; Jurkiewicz et al., 2006; Mole et al., 2014; Wrigley et al., 2009). Functional MRI showed the brain reorganization patterns after SCI appeared to be dynamic and influenced by the level, completeness, duration, and the extent of clinical recovery (Kokotilo et al., 2009). Similarly, the varying injury level and severity, wide range of disease duration, prolonged exposure to rehabilitation training and medicine might contribute to the structural inconsistencies across previous studies.

Unfortunately, given that the study of structural changes in human SCI lacks pre‐injury data, includes small samples, and is associated with difficulties in follow‐up (Nardone et al., 2018), most studies have mainly focused on the volumetric comparison between SCI patients and healthy controls. Thus, it remains largely unknown whether cortical morphometric changes occur within different SCI patient populations (e.g., complete or incomplete SCI, tetraplegia, or paraplegia). Chen et al. (2017) have attempted to explore the difference in the gray matter volume (GMV) between complete and incomplete SCI subgroups; however, no significant difference was found. The small sample of each SCI subgroup may contribute to the result. A study investigated the volumetric changes in a small sample of SCI patients showed higher levels of injury resulting in greater loss of GMV (Karunakaran et al., 2019), which indicated that injury level may affect GMV in the brain. Two longitudinal studies that quantified volumetric changes found that cortical white and gray matter atrophy progressed over one year and two years (Freund, Weiskopf, et al., 2013; Ziegler et al., 2018), which suggested that the disease duration may play an important role in the neurodegenerative pathology changes of the structure and function at the brain level.

To address these questions, the purpose of this study was to explore the difference in the brain GMV between SCI patients and healthy controls, tetraplegia and paraplegia, as well as complete and incomplete SCI patients using voxel‐based morphometry (VBM) analysis. Furthermore, we attempted to explore the patterns of whole‐brain structural changes as the disease progresses after SCI and determine whether these patterns involve time‐dependent anatomical changes. Finally, we aimed to investigate the correlations between regional patterns of brain change and clinical variables. We hypothesized that (a) there exist brain GMV differences between SCI subgroups; (b) GMV changes as the duration progress; (c) there exists a clinical relevance between the altered GMV and motor or sensory scores.

## 2 MATERIALS AND METHODS

### 2.1 Subjects

**FIGURE 1:**
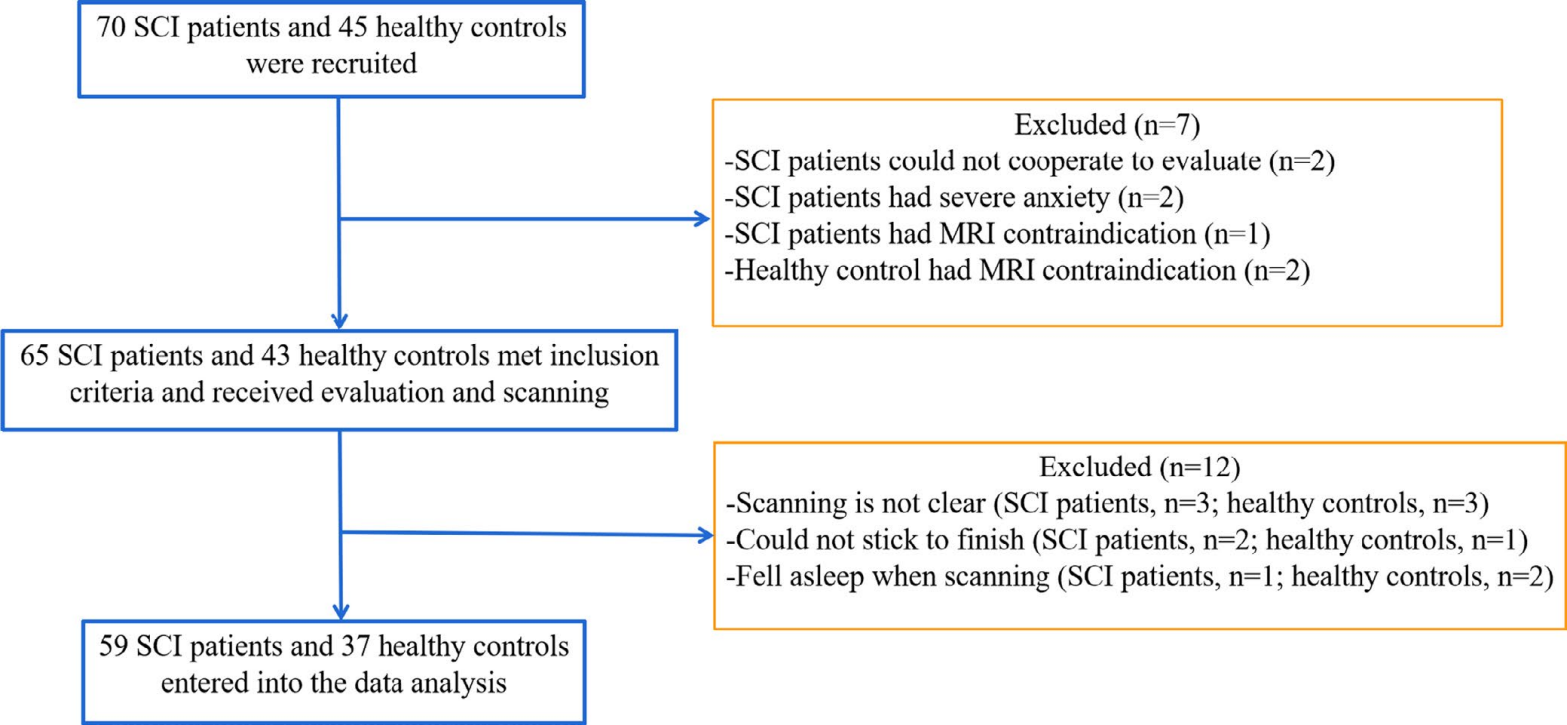
Study flow diagram. SCI, spinal cord injury

In the current study, patients with SCI were recruited consecutively from the Department of Spinal and Neural Functional Reconstruction at China Rehabilitation Research Center (Beijing, China) from July 2017 to May 2019. All SCI participants have previously received rehabilitation therapy. Seventy subjects with SCI and 50 healthy controls were recruited in total. Of them, 65 SCI patients and 43 healthy controls completed MRI, and an additional six SCI subjects and six healthy controls were excluded due to head motion or falling asleep during MRI. Finally, 59 right‐handed SCI patients (49 males and 10 females, mean age 38.24 ± 11.27 years, ranging from 18 to 60 years) and 37 right‐handed healthy controls (24 males and 13 females, mean age 39.81 ± 8.98 years, ranging from 18 to 60 years) entered into the data analysis (Figure 1).

All the SCI patients had no concomitant brain injury associated with the trauma leading to the SCI. No patient suffered from a psychiatric disorder or other neurological diseases except SCI. All patients had no MRI contraindications. All patients underwent a comprehensive clinical assessment using the classification scale of the American Spinal Injury Association (ASIA), including the ASIA Impairment Scale, total motor score (i.e., upper + lower motor score), and total sensory score (i.e., light touch + pinprick score). We also evaluated psychological factors regarding anxiety and depression in all subjects before scanning using Hamilton Anxiety Rating Scale (HAMA) and Hamilton Depression Rating Scale (HAMD).

These healthy controls responding to the poster were not patients within the hospital. None of the controls had seizure, medical or mental illnesses, or contraindications to MRI. The age and years of education between the healthy controls and SCI patients were not significantly different.

All subjects provided written informed consent before the study, which was approved by the medical ethics committee of China Rehabilitation Research Center (Beijing, China) (ref: 2017‐071‐1).

### 2.2 Anatomical imaging of the brain

All subjects were scanned on a 3‐T MRI scanner (Philips Ingenia, Best, The Netherlands) at China Rehabilitation Research Center, Beijing, China. Weiyong Yu, who was the doctor from the Department of Radiology at the hospital, performed MRI. A high‐resolution structural T1‐weighted anatomic sequence was acquired in a sagittal orientation using a 3‐D gradient echo based sequence (T1W‐3D‐TFE‐ref) with the following parameters: repetition time (TR) = 7.64 ms; echo time (TE) = 3.73 ms; flip angle (FA) = 8°; number of slices = 180; slice thickness = 2 mm; field of view (FOV) = 256 × 256 mm^2^; matrix = 256×256; and isotropic voxel = 1×1 × 1 mm^3^.

### 2.3 Data processing and VBM analysis

T1‐weighted image preprocessing was performed using CAT12 in Statistical Parametric Mapping (SPM12) software (https://www.fil.ion.ucl.ac.uk/spm/software/spm12/) implemented in MATLAB 2013b (Math Works). The following steps were included: First, all T1‐weighted images were manually reoriented to place the anterior commissure at the origin of the 3D Montreal Neurological Institute (MNI) space. Second, the structural images were segmented into the gray matter (GM), white matter (WM), and cerebrospinal fluid (CSF) using the unified standard segmentation option in SPM12. Third, the GM was warped into an optimal (average) space using Diffeomorphic Anatomical Registration with the Exponentiated Lie algebra (DARTEL) toolbox. Forth, the resulting GM images were then modulated and affine‐transformed to the MNI space and smoothed with a 12‐mm full‐width at half‐maximum Gaussian kernel.

### 2.4 Region of interest analyses of GMV

To investigate associations between brain anatomical changes and clinical symptoms in exploratory studies, we performed a region of interest (ROI) analysis of the GMV. ROI analyses were conducted by averaging the GMV values of voxels. The regions that exhibited alterations in the GMV when the SCI patients were compared to the healthy controls and between the SCI subgroups were defined as ROIs, including the middle frontal gyrus (MiFG), superior frontal gyrus (SFG), medial frontal gyrus (MeFG), primary motor cortex (M1), primary somatosensory cortex (S1), thalamus. We also extracted values of prior ROIs reported abnormal in patients with SCI in previous studies (Chen et al., 2017; Hou et al., 2014; Mole et al., 2014), including the superior temporal gyrus, supplementary motor area (SMA), pallidum, and cuneus. The ROIs were generated using the WFU pickatlas toolbox. Each ROI of each subject was subsequently extracted.

### 2.5 Statistical analysis

Voxel‐wise comparisons of the GMV between the SCI patients and healthy controls were performed using two‐sample *t* tests (voxel‐level uncorrected *p* < .001, nonstationary cluster‐level family‐wise error (FWE) correction with *p* < .05). Voxel‐wise comparisons of the GMV were also performed using two‐sample *t* tests between complete SCI and incomplete SCI subgroups, as well as between tetraplegia and paraplegia subgroups (voxel‐level uncorrected *p* < .001, nonstationary cluster‐level FWE correction with *p* < .05). Age, gender, years of education, and total intracranial volume (TIV) (i.e., CSF + WM + GMV) were modeled as covariates of no interest in all *t* tests. Partial correlation analyses were performed to explore the associations between the GMV and injury duration and clinical variables in patients with SCI and between the GMV of each subgroup (complete SCI and incomplete SCI, and tetraplegia and paraplegia) after removing age, gender, years of education, and TIV effects. Continuous clinical variables were tested using two‐tailed *t* tests, while gender differences were examined by chi‐square tests. *p* < .05 were considered to be statistically significant. Statistical analyses were carried out in SPM software (https://www.fil.ion.ucl.ac.uk/spm/software/spm12/) and SPSS version 20 (SPSS Inc.).

## 3 RESULTS

### 3.1 Demographic and clinical characteristics

The clinical and demographic data of all subjects are shown in Table 1. There were 59 right‐handed SCI patients (49 males and 10 females, mean age 38.24 ± 11.27 years) and 37 right‐handed healthy controls (24 males and 13 females, mean age 39.81 ± 8.98 years) in this study. All patients suffered from SCI due to trauma, including vehicle accident (13 cases), fall (22 cases), crush by weight (19 cases), sport injury (three cases), and others (two cases). Forty‐one patients had a complete lesion and 18 patients had an incomplete lesion based on the ASIA impairment classification. There were 13 patients with tetraplegia and 46 patients with paraplegia based on the injury level in the SCI group. In the SCI patients, the range of the disease duration was from 1 month to 18 years, with a mean of 24.9 ± 51.97 months. The score of HAMA in healthy control was 19.94 ± 5.8, while the score was 20.75 ± 6.4 in SCI group. And the score of HAMD in healthy control was 8.75 ± 6.1, while the score was 7.25 ± 4.3 in SCI group. Difference did not reach the statistical significance between healthy controls and SCI patients and between SCI subgroups in HAMA and HAMD. No statistically significant differences were found between the SCI patients and healthy controls in age and education (*p* > .05). There was a difference between the SCI patients and healthy controls in gender (*p* = .04).

**TABLE 1:** Demographic and clinical characteristics of SCI patients and healthy controls

Characteristics	SCI (*n* = 59)	Healthy controls (*n* = 37)	*p*‐Value	Complete SCI (*n* = 41)	Incomplete SCI (*n* = 18)	*p*‐Value	Tetraplegia (*n* = 13)	Paraplegia (*n* = 46)	*p*‐Value
Age, years, mean ± *SD*	38.24 ± 11.27	39.81 ± 8.98	.48	38.76 ± 11.22	37.06 ± 11.62	.60	42.17 ± 9.59	37.82 ± 11.42	.23
Gender, male:female	49:10	24:13	.04	38:3	13:5	.04	11:2	40:6	.83
Education, years, mean ± *SD*	11.25 ± 2.90	11.38 ± 3.88	.86	10.95 ± 3.02	11.94 ± 2.58	.23	10.83 ± 2.21	11.22 ± 3.03	.68
Total motor scores, mean ± *SD*	50.32 ± 17.13	100.00 ± 0.00	<.001	45.56 ± 12.40	61.17 ± 21.45	<.001	36.08 ± 24.68	52.40 ± 13.68	<.001
Total sensory scores, mean ± *SD*	128.54 ± 45.80	224.00 ± 0.00	<.001	122.66 ± 42.50	141.94 ± 51.33	.14	69.58 ± 37.32	139.42 ± 37.16	<.001
Upper motor scores, mean ± *SD*	44.53 ± 11.75	50.00 ± 0.00	<.001	44.80 ± 11.90	43.89 ± 11.71	.79	27.58 ± 11.67	47.60 ± 8.34	<.001
Lower motor scores, mean ± *SD*	6.27 ± 12.16	50.00 ± 0.00	<.001	0.76 ± 2.05	18.83 ± 15.93	<.001	8.50 ± 15.66	5.36 ± 10.97	.42
Light touch scores, mean ± *SD*	65.95 ± 23.58	112.00 ± 0.00	<.001	61.56 ± 21.16	75.94 ± 26.28	.03	41.17 ± 21.54	71.12 ± 19.65	<.001
Pinprick scores, mean ± *SD*	64.22 ± 24.13	112.00 ± 0.00	<.001	61.15 ± 21.41	71.22 ± 28.87	.14	28.42 ± 18.65	70.22 ± 19.36	<.001
Injury time, months, mean ± *SD*	34.90 ± 51.97	0.00 ± 0.00	<.001	35.78 ± 50.78	32.89 ± 56.05	.85	34.33 ± 40.38	33.30 ± 53.66	.95
Score of HAMA, mean ± *SD*	20.75 ± 6.40	19.94 ± 5.80	.76	20.15 ± 6.11	19.96 ± 5.40	.87	21.05 ± 5.84	20.85 ± 6.34	.81
Score of HAMD, mean ± *SD*	7.25 ± 4.30	8.75 ± 6.10	.62	7.32 ± 4.12	8.05 ± 3.30	.55	7.25 ± 4.20	7.75 ± 4.35	.93
Etiology, no.									
Vehicle accident	13	—	—	8	5	—	3	10	—
Fall	22	—	—	15	7	—	5	17	—
Crush by weight	19	—	—	14	5	—	4	15	—
Sport injury	3	—	—	1	2	—	1	2	—
Others	2	—	—	2	0	—	0	2	—

Abbreviations: no., number; SCI, spinal cord injury.

### 3.2 Whole‐brain VBM analysis

**FIGURE 2:**
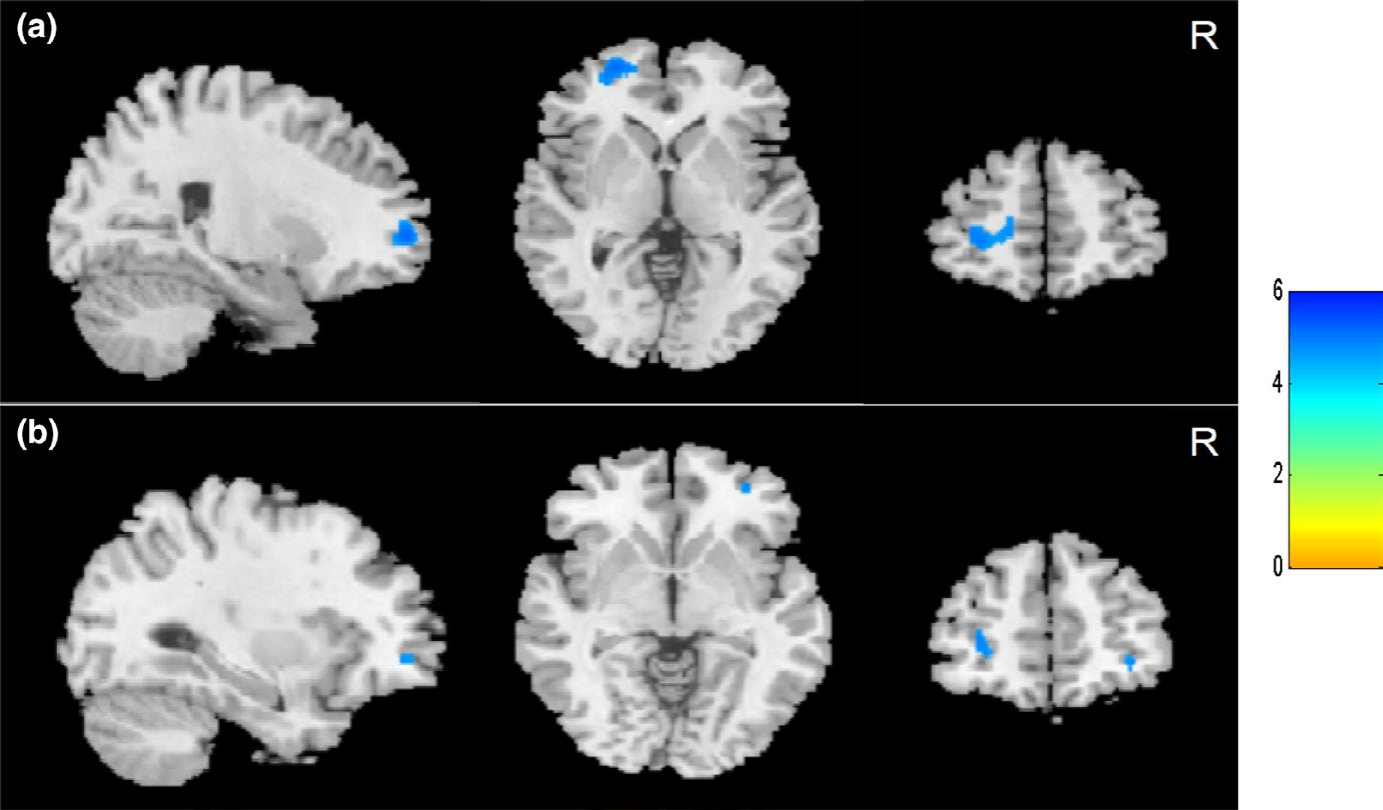
Cortical gray matter volume reduction in spinal cord injury (SCI) patients compared to healthy controls. (a) Smaller brain gray matter volume in left frontal gyrus and (b) reduced gray matter volume in right middle frontal gyrus in SCI patients (voxel level, *p* < .001, uncorrected; cluster level, *p* < .05, family‐wise error corrected for multiple comparisons). Colour bar graph represents T value. R, right

**FIGURE 3:**
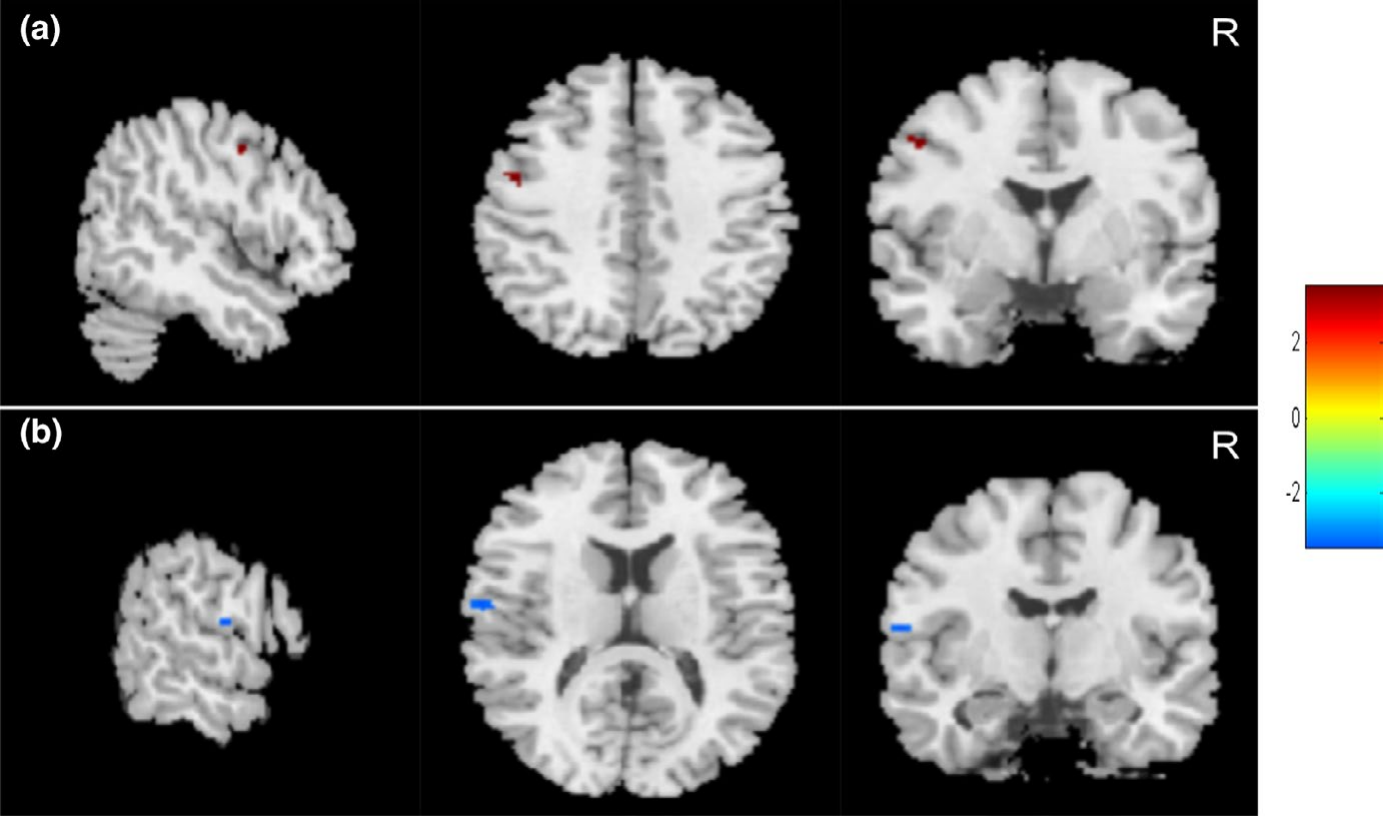
Cortical gray matter volume changes in patients with complete spinal cord injury (SCI) compared to incomplete SCI. (a) Increased brain gray matter volume in left primary motor cortex (red area) and (b) reduced gray matter volume in left primary somatosensory cortex (blue area) (voxel level, *p* < .001, uncorrected; cluster level, *p* < .05, family‐wise error corrected for multiple comparisons) in complete SCI patients compared to incomplete SCI patients. Colour bar graph represents *T* value. R, right

**FIGURE 4:**
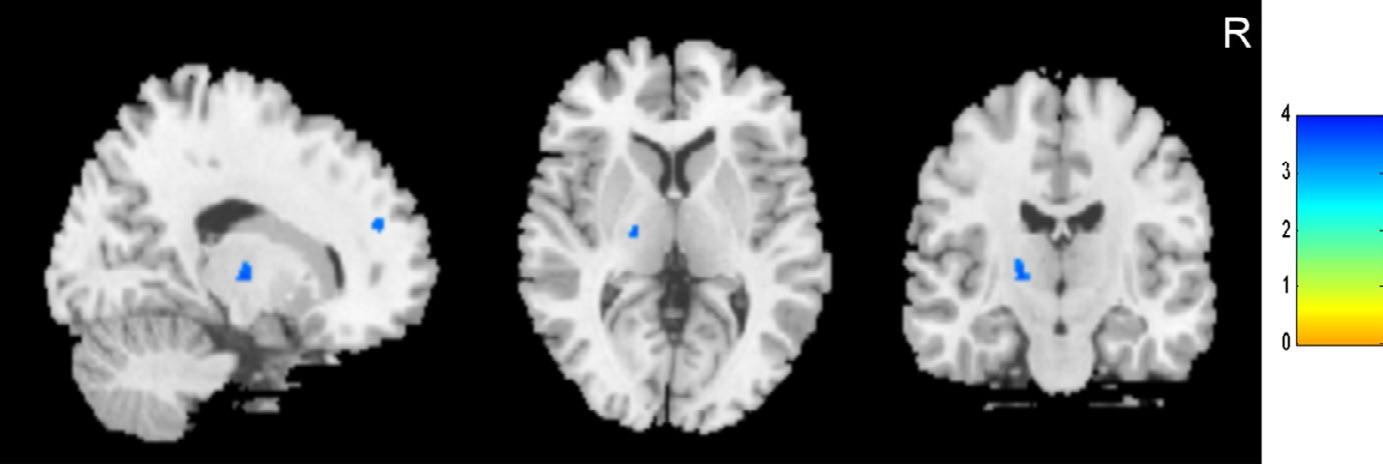
Cortical gray matter volume decreased in left thalamus and left superior frontal gyrus in individuals with tetraplegia compared to paraplegia (voxel level, *p* < .001, uncorrected; cluster level, *p* < .05, family‐wise error corrected for multiple comparisons). Colour bar graph represents T value. R, right

Compared to the healthy controls, the SCI patients showed significantly lower GMV in the bilateral MiFG, left SFG, and left MeFG (voxel‐level uncorrected *p* < .001, FWE‐corrected for multiple comparisons *p* < .05; Figure 2 and Table 2). When the complete SCI patients were compared to the incomplete SCI patients, a lower GMV was found in the left S1, with higher GMV in the left M1 (voxel‐level uncorrected *p* < .001, FWE‐corrected for multiple comparisons *p* < .05; Figure 3 and Table 3). A lower cortical GMV was found in the left thalamus and left SFG in the tetraplegia patients than in the paraplegia (voxel‐level uncorrected *p* < .001, FWE‐corrected for multiple comparisons *p* < .05; Figure 4 and Table 4).

**TABLE 2:** Gray matter volume loss in patients with SCI compared to healthy controls

No. of cluster	Voxel sizes	Anatomical region	MNI coordinate			Volume change	Peak *T*‐value	*p*‐Value (FWE‐corrected)	*p*‐Value (uncorrected)
			*X*	*Y*	*Z*				
1	28	MiFG _R	32	50	−5	Decreased	−4.83	.027	<.001
2	232	SFG_L	−27	57	5	Decreased	−5.3	.005	<.001
	201	MiFG_L							
	57	MeFG_L							

Abbreviations: L, left; MeFG, medial frontal gyrus; MiFG, middle frontal gyrus; R, right; SFG, superior frontal gyrus.

**TABLE 3:** Difference in cortical gray matter volume in complete SCI patients compared to incomplete SCI patients

No. of cluster	Voxel sizes	Anatomical region	MNI coordinate			Volume change	Peak *T*‐value	*p*‐Value (FWE‐corrected)	*p*‐Value (uncorrected)
			*X*	*Y*	*Z*				
1	33	M1_L	−50	−2	42	Increased	3.53	.035	<.001
2	27	S1_L	−63	−8	14	Decreased	−3.38	.04	.001

Abbreviations: L, left; M1, primary motor cortex; MiFG, middle frontal gyrus; R, right; S1, primary somatosensory cortex.

**TABLE 4:** Difference in cortical gray matter volume in patients with tetraplegia compared to paraplegia

No. of cluster	Voxel sizes	Anatomical region	MNI coordinate			Volume change	Peak *T*‐value	*p*‐Value (FWE‐corrected)	*p*‐Value (uncorrected)
			*X*	*Y*	*Z*				
1	39	Thalamus_L	−18	−15	2	Decreased	−3.65	.024	<.001
2	39	SFG_L	−20	47	21	Decreased	−3.51	.032	<.001

Abbreviations: L, left; SFG, superior frontal gyrus.

### 3.3 ROI analysis

**FIGURE 5:**
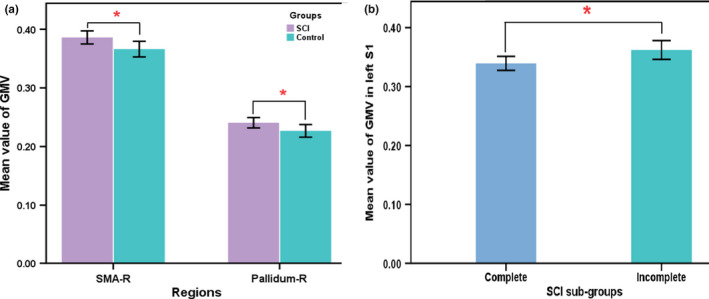
Difference of ROI analyses between SCI patients and healthy controls (a) and SCI sub‐groups (b). SCI, spinal cord injury; GMV, gray matter volume; SMA, supplementary motor area; S1, primary somatosensory cortex; R, right. * with statistical significance *p* < .05

The ROI analysis showed the GMV increased in the right SMA and right pallidum in the patients with SCI compared to the healthy controls (uncorrected voxel‐level *p* < .01, cluster‐level correction with *p* < .05; Figure 5a). The GMV in the left S1 in the complete SCI patients was lower than in the incomplete SCI patients (uncorrected voxel‐level *p* < .01, cluster‐level correction with *p* < .05; Figure 5b). There was no significant difference in the regional GMV between the tetraplegia and paraplegia patients (uncorrected voxel‐level *p* < .01, cluster‐level correction with *p* < .05).

### 3.4 Correlation analysis

**FIGURE 6:**
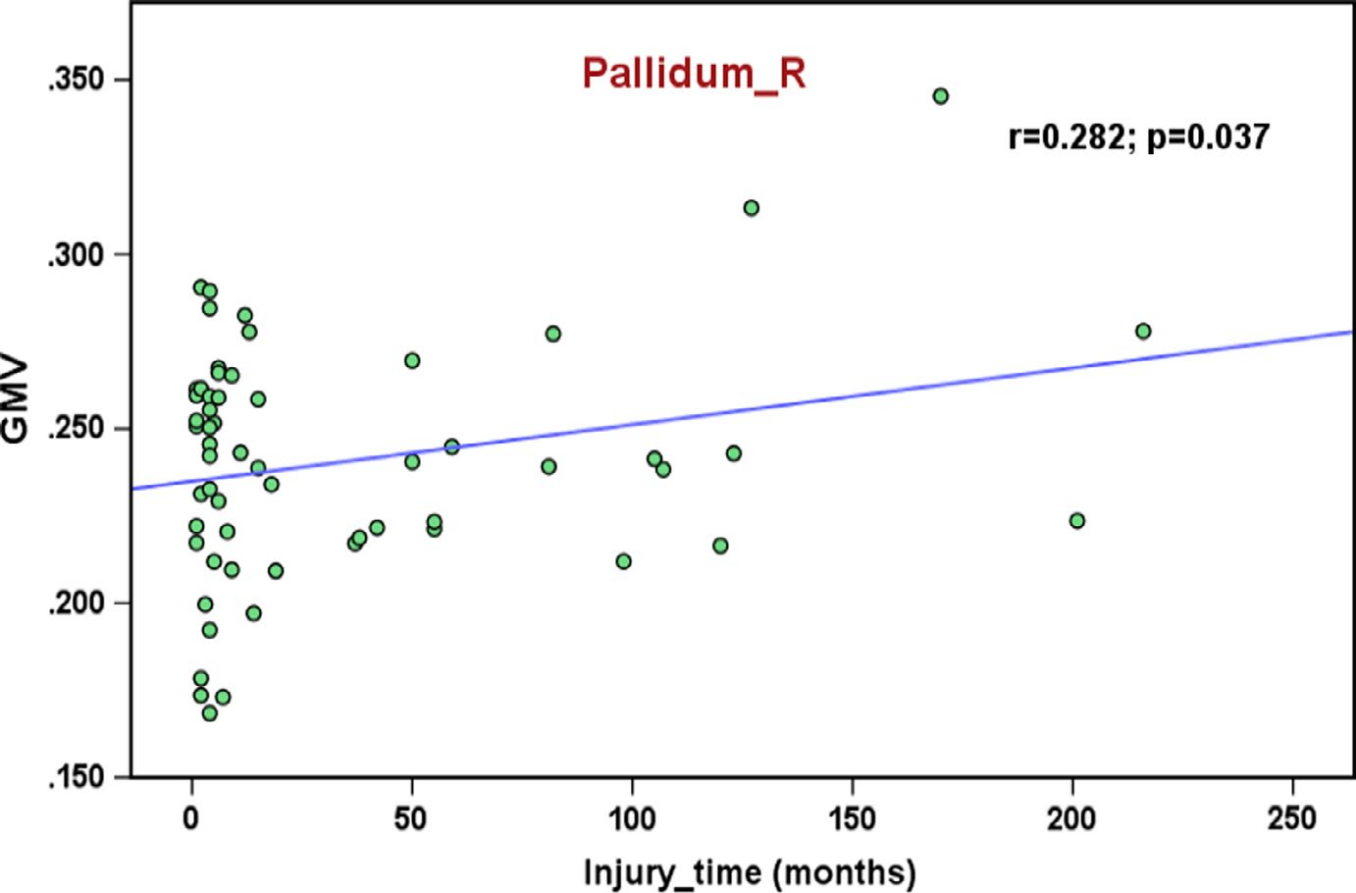
Correlation of injury time with GMV in right pallidum in patients with SCI. GMV, gray matter volume; R, right

**FIGURE 7:**
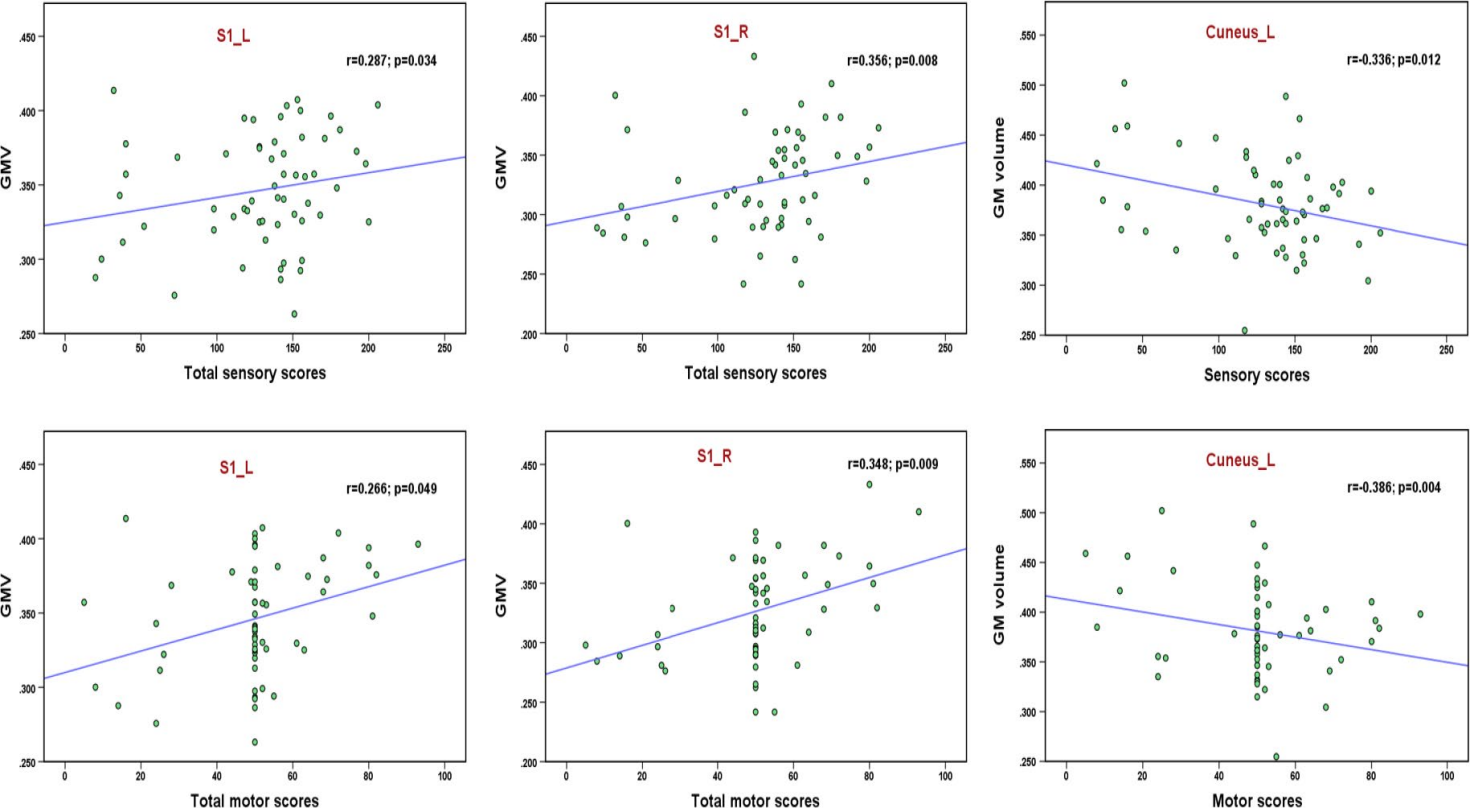
Correlation of total sensory, motor scores with GMV in bilateral S1 and left cuneus in patients with SCI. GMV, gray matter volume; S1, primary somatosensory cortex; L, left; R, right

The duration was positively correlated with the GMV in the right pallidum (*r* = 0.282, *p* = .037; Figure 6). The participant with GMV = 350 was not an outlier, due to the exclusion from the analysis did not significantly change our findings. Positive correlations were observed between the GMV in the bilateral S1 and the total ASIA motor and sensory scores (Figure 7). The GMV in the left cuneus was negatively correlated with the total motor and sensory scores in the patients with SCI (Figure 7).

## 4 DISCUSSION

In the present study, we found brain GMV changes following SCI affected by the injury severity, cervical and thoracic levels, and duration. In general, the GMV decreased in the frontal area and increased right SMA and right pallidum in the patients with SCI compared to the healthy controls. There were significant differences in the GMV (left M1 and S1) between the complete SCI and incomplete SCI subgroups, as well as differences (left thalamus and SFG) between the patients with tetraplegia and paraplegia subgroups. Moreover, a positive correlation was found between the GMV of the right pallidum and the injury duration. Finally, we found positive associations between the GMV in bilateral S1 and the total motor and sensory scores in the patients with SCI; the GMV in the left cuneus of the patients showed a negative correlation with the motor and sensory scores. These findings in our study provide further insights into the cortical structural changes following SCI.

Compared to healthy controls, the brain GMV decreased in SCI patients mainly in the frontal gyrus in the whole‐brain VBM analysis, including the MiFG, left SFG, and left MeFG. Moreover, the ROI analysis showed increased GMV in the right SMA and right pallidum in patients with SCI. Our results support that significant brain structural changes in the human brain after SCI, which is consistent with previous studies (Chen et al., 2017; Freund et al., 2011; Hou et al., 2014; Jurkiewicz et al., 2006; Wrigley et al., 2009). However, the decreased brain GMV shown in most previous studies was localized in the sensorimotor cortex, such as M1 and S1. Freund et al. (2011) reported GMV decreased in the leg area of M1 and S1 after SCI. Hou et al. (2014) found SCI patients had significant GM atrophy in the M1, S1, SMA, and thalamus in the early stage of SCI. Jurkiewicz et al. (2006) observed SCI subjects had reduced GMV in the bilateral S1. Unfortunately, we did not identify volumetric changes in S1, M1, or S2 in the present study. But our findings were in accord with several studies (Chen et al., 2017; Crawley et al., 2004; Yoon et al., 2013). Nardone et al. (2018) given an potential explanation is that the presence of an extensive array of collateral connections within M1 and connections between M1 and higher order motor areas may maintain cellular activity in the motor system, possibly reducing or preventing corticospinal neuronal atrophy after axonal injury. Furthermore, as the SCI patients in our study received rehabilitation training all the time since injury, the rehabilitation effect on cortical reorganization after SCI might play an important role in the results. As Villiger et al. (2015) reported that structural plasticity at the cortical and brainstem level after virtual reality‐augmented training in incomplete SCI patients.

In the present study, we found lower GMV in the frontal area, including MiFG, SFG, and MeFG. The MiFG is involved in the performance of executive function and emotion regulation (John et al., 2006; Markov et al., 2009; Ohira et al., 2006). The SFG has been reported to be involved in various cognitive and motor control tasks (Li et al., 2013). The MeFG is a region associated with high‐level executive functions and decision‐related processes (Talati & Hirsch, 2005). Thus, these three regions are involved in executive and cognitive processes in general. Structural changes in the frontal area in patients may be linked to the sensorimotor deficits and mental state after SCI. Craig et al. (2017) reported cognitive impairment and negative mood states in patients with SCI. So, more attention on cognitive performance needs to be given in the future in these populations.

Although most neurons in the brain are not directly injured as a result of SCI, the efferent motor and afferent sensory pathways are severely disrupted. Why did structural changes with GM atrophy occur in remote brain areas? Early study (Barron et al., 1988) indicated that pyramidal neurons in layer Vb of the rat sensorimotor cortex do not die but atrophy, and the neuronal atrophy is unaccompanied by ultrastructural alterations. A study examined the disruption of the corticospinal tract in two macaque monkeys and demonstrated that the vast majority of the axotomized corticospinal neurons did not degenerate. Rather, their somata shrank, compared to the opposite hemisphere or intact monkeys (Wannier et al., 2005). Huber et al. (2015) reported that the volume reduction at the cortical level reflects soma shrinkage of injured corticomotor neurons. In contrast, corticospinal neuron death has been reported in animal research. Hains et al. (2003) found apoptotic cell death in the somatosensory cortex after spinal cord transection, which resulted in the apoptosis of up to 40% of M1 corticospinal neurons. Another study (Kim et al., 2006) reported morphometric changes in the M1 synaptic spine density after SCI in rats. Moreover, several studies suggested that these changes may be caused by direct or secondary Wallerian degeneration. Freund, Curt, et al. (2013) proposed that GM in the sensorimotor cortex of the brain atrophied after SCI might be caused by demyelination of axons and atrophy of neuronal cell bodies. However, the underlying mechanisms responsible for cortical GM atrophy are not fully understood in humans because imaging studies could not provide information regarding the pathological processes leading to atrophy in the brain.

The majority of structural studies typically focus on the comparison of potential differences between SCI patients and healthy controls. Few studies have reported brain structural differences between patients with complete and incomplete SCI. Chen et al. (2017) showed that no significant difference was found in the GMV between complete and incomplete SCI subgroups. The small sample of each SCI subgroup may contribute to the findings. In the present study, we found lower GMV in the left S1 and higher GMV in the left M1 and left SFG in complete SCI patients than in incomplete SCI patients. It is known that sensory function is completely interrupted between the brain and below injury level, while it is reserved to a certain degree in incomplete SCI patients. The input information was restrained due to the injury; thus, it is easy to explain the lower GMV in the left S1 in complete SCI patients.

Surprisingly, a higher GMV in the left M1 in complete SCI patients was found in our study. To investigate the potential mechanism in this phenomenon, we analyzed the characteristics of the patients in the subgroup with complete SCI and found that 83% (34/41) were paraplegia who had slightly better hand motor function than the patients with incomplete SCI (44.80 ± 11.90 vs. 43.89 ± 11.71). Thus, most patients reserved hand function even they were complete SCI. A functional MRI study showed a similar finding in which spinal cord compression resulted in an increase in the volume of activation (VOA) within the precentral gyrus and a loss of VOA within the postcentral gyrus compared to controls (Duggal et al., 2010). However, these phenomena regarding different changes in the GMV in M1 and S1 in complete and incomplete SCI patients require further study.

There is a substantial difference in upper limb movements between individuals with paraplegia and tetraplegia. Patients with paraplegia have intact arms and hands, whereas those with tetraplegia have some degree of deficits in the upper limbs. Functional MRI studies have shown different motor cortex activation between individuals with paraplegia and tetraplegia. For example, Curt et al. (2002) found greater activation in the contralateral sensorimotor cortex, contralateral thalamus, bilateral cerebellum, and ipsilateral parietal cortex in individuals with complete paraplegia than in controls, while complete tetraplegia showed substantially less brain activation in motor areas than controls. Sabre et al. (2016) demonstrated when patients with thoraco‐lumbar traumatic SCI moved their hand, the VOA in the contralateral M1 was significantly larger among the traumatic SCI patients who did not recover than in the controls. However, the VOA did not enlarge during the ankle movements. They thought that the increased cortical activation in SCI patients may be caused by the increased use of the upper limbs. Similarly, cortical structural changes after SCI might be different between paraplegia and tetraplegia due to the use of the upper limbs.

Most structural studies have focused on patients with cervical SCI, thoracic SCI, or mixed with cervical and thoracic SCI. To date, only few studies have directly compared brain structural differences between patients with paraplegia and tetraplegia. In this study, we found lower GMV in the left thalamus and left SFG in patients with tetraplegia than in paraplegia in the whole‐brain analysis. In general, the thalamus is considered an important relay center that transmits and processes sensory and motor signals to the cerebral cortex (Haber & Calzavara, 2009; Sommer, 2003). Our findings of anatomical abnormalities in the left thalamus in tetraplegia patients may be associated with their worse sensory and motor function compared to paraplegia. The human SFG consists of multiple dissociable subregions that have distinct connection patterns, and these subregions are involved in different functional networks. Specifically, the posterior SFG was connected with the precentral gyrus, caudate, thalamus, and frontal operculum, which are nodes of the motor control network (Li et al., 2013). The infiltration of the posterior part of the SFG by a lesion disturbs some complex hand motor functions (Martino et al., 2011). Therefore, the SFG plays an important role in hand motor control. Hand motor function in patients with tetraplegia is worse than that in paraplegia. Thus, the lower GMV of the SFG in tetraplegia might be driven by the worse hand motor function.

Region of interest analysis showed increased GMV in right SMA and right pallidum in SCI patients compared to healthy controls. We also found a positive correlation between the GMV of the right pallidum and the injury duration in SCI patients, which is inconsistent with the findings of previous studies (Chen et al., 2017; Freund et al., 2011; Ziegler et al., 2018). For example, Chen et al. (2017) found a negative trend for the correlation between the right orbital frontal cortex (OFC) GMV of patients with SCI and the injury duration and a significant negative correlation between the right OFC GMV of the subacute subgroup and the injury duration. Several studies have shown that the brain structure or function could be influenced by intervention during the duration of extensive disease following SCI (Curt, Alkadhi, et al., 2002; Jurkiewicz et al., 2007; Lundell et al., 2011; Villiger et al., 2015). The positive correlation in our study could be related to the fact that our patients were all inpatients who received rehabilitation in the hospital since injury. Freund's team reported that cortical white and GM atrophy progressed over 1 and 2 years (Freund, Weiskopf, et al., 2013; Ziegler et al., 2018). The disease course is limited to the first two years after injury, while the duration of our patients lasted for 20 years; thus, brain plasticity might have occurred to compensate for the motor and sensory deficits. Sabre et al. (2016) reported that their patients in the chronic phase of thoracic or lumbar level traumatic SCI had increased brain activation when they moved their intact limb. This finding indicates that the longer the time the patients have trained their hands, the larger the activation of the cortex will become.

To date, few studies have explored the correlation between GM changes and clinical variables. In the present study, the GMV in the bilateral S1 of patients showed a positive correlation with the total sensory and motor scores. The findings indicated that a better clinical performance was associated with a greater GMV in S1, which is consistent with the findings of previous studies. Hou et al. (2014) observed that the GMV in M1 was positively correlated with the total ASIA motor score in patients with SCI. Chen et al. (2017) found the right OFC GMV was positively related to the left motor score in patients with SCI. Moreover, the GMV in the left cuneus was negatively correlated with the total motor and sensory scores in patients with SCI, which suggested that a worse clinical performance was associated with a greater GMV in the left cuneus. Cuneus areas are classically related to visual information processing; in particular, the anteromedial cuneus has the temporal position needed to interact with the primary visual cortex (V1) and thereby modify information transferred via V1 to extrastriate cortices (Vanni et al., 2001). The brain network reorganization showed that the visual cortex became less connected with the M1 but more connected with the cingulate gyrus and parietal lobe, which suggests an enhancement in visual‐related sensory processing after the loss of spinal afferent (Hawasli et al., 2018). Therefore, the left cuneus might play an important role in compensating for sensory dysfunction by enhancing visual function in patients with SCI.

Several limitations should be considered in the present study. Despite the large number of SCI patients enrolled in the study, there were relatively smaller samples in the incomplete SCI and tetraplegia subgroups than in the complete SCI and paraplegia subgroups. Moreover, there was a little difference in gender between the healthy controls and SCI patients. Finally, the exact histopathological processes that lead to changes in volumetrics over time are complex, and basic researches and longitudinal clinical studies will be useful in the future.

## 5 CONCLUSIONS

In conclusion, our study provides imaging evidence for brain structural abnormalities in patients with SCI and significant differences in complete/incomplete and paraplegia/tetraplegia subgroups. Furthermore, the positive time‐dependent changes of GMV in the right pallidum indicate that a longer disease course may not lead to more severe GM atrophy and that a rehabilitation effect on cortical reorganization after SCI may play an important role in the results. These results suggest brain structural changes occur after SCI and that these changes may depend on the injury severity, injury level, and injury duration.

## CONFLICT OF INTEREST

The authors declare that they have no potential conflicts of interest.

## AUTHOR CONTRIBUTIONS

Li JJ, Guo Y, and Gao F conceived and designed the study. Yang ML, Du LJ, and Yang DG helped in designing the study for better performance. Guo Y and Gao F performed the study. Guo Y wrote the manuscript. Yu WY and Chen ZB performed MRI scanning. Guo Y and Guo H performed data preprocessing and statistical analysis.

### Peer Review

The peer review history for this article is available at https://publons.com/publon/10.1002/brb3.2037.

## Data Availability

All the data supporting our findings are contained in the manuscript. The datasets used and/or analyzed in the current study are available from the corresponding author on reasonable request.
